# Influence of Head and Neck Position on Oropharyngeal Leak Pressure and Cuff Position with the ProSeal Laryngeal Mask Airway and the I-Gel: A Randomized Clinical Trial

**DOI:** 10.1155/2015/705869

**Published:** 2015-01-11

**Authors:** Sandeep Kumar Mishra, Mohammad Nawaz, M. V. S. Satyapraksh, Satyen Parida, Prasanna Udupi Bidkar, Balachander Hemavathy, Pankaj Kundra

**Affiliations:** Department of Anaesthesiology and Critical Care, Jawaharlal Institute of Postgraduate Education and Research (JIPMER), Pondicherry 605006, India

## Abstract

*Background*. This study was designed to assess and compare the effect of head and neck position on the oropharyngeal leak pressures and cuff position (employing fibreoptic view of the glottis) and ventilation scores between ProSeal LMA and the I-gel. *Material and Methods*. After induction of anesthesia, the supraglottic device was inserted and ventilation confirmed. The position of the head was randomly changed from neutral to flexion, extension, and lateral rotation (left). The oropharyngeal leak pressures, fibreoptic view of glottis, ventilation scores, and delivered tidal volumes and end tidal CO_2_ were noted in all positions. *Results*. In both groups compared with neutral position, oropharyngeal leak pressures were significantly higher with flexion and lower with extension but similar with rotation of head and neck. However the oropharyngeal leak pressure was significantly higher for ProSeal LMA compared with the I-gel in all positions. Peak airway pressures were significantly higher with flexion in both groups (however this did not affect ventilation), lower with extension in ProSeal group, and comparable in I-gel group but did not change significantly with rotation of head and neck in both groups. *Conclusion*. Effective ventilation can be done with both ProSeal LMA and I-gel with head in all the above positions. ProSeal LMA has a better margin of safety than I-gel due to better sealing pressures except in flexion where the increase in airway pressure is more with the former. Extreme precaution should be taken in flexion position in ProSeal LMA.

## 1. Introduction

Supraglottic devices have been used in different head and neck positions for various surgeries [1–4]. ProSeal LMA (LMA North America, San Diego, CA, USA) and I-gel (noninflatable cuff) (Intersurgical Ltd., Wokingham, UK) are two prototype devices with channels for insertion of gastric tubes. The cuffs of both devices sit in the pharynx and form a seal for ventilation and also possibly for prevention of aspiration from above. Due to changes in the shape of the pharynx [5] during head and neck movement, there is a possibility of changes in the force transmitted to the cuff along the airway tube during ventilation. Previous studies have shown evidence of changes in the efficacy of seal and also displacement during changes in the head and neck position [6, 7]. The primary objectives of this study were to compare the oropharyngeal leak pressures of I-gel and the ProSeal LMA at different head and neck positions, namely, neutral, flexion, extension, and left rotation. We also assessed and compared the fiberoptic view of glottis and ventilator score of both devices in different head and neck positions (secondary objectives).

## 2. Materials and Methods

After approval from the Institutional Ethics Committee (JIP/IEC/SC/2012/3/5 22.05.2012), this study was conducted in sixty ASA physical status I and II patients aged 18 to 65 years of either sex scheduled for elective surgeries at Jawaharlal Institute of Postgraduate Education and Research (JIPMER), Pondicherry, India. This study is also enrolled in a Clinical Trials Registry (CTRI India) (REF/2014/05/006899 and CTRI/2014/09/004961). Patients at risk of aspiration, any pathology of the neck, upper respiratory tract infection, anticipated difficult airway, body mass index > 35 kg/m^2^, history of obstructive sleep apnea, history of lung diseases, potentially full stomach, or having a history of gastroesophageal reflux were excluded from the study. The patients were randomized to either I-gel group or ProSeal LMA group by computer generated allocation. Written informed consent was obtained from all patients recruited for the study. On the night before surgery, all patients were premedicated with tablet famotidine 20 mg, tablet diazepam 10 mg, and tablet metoclopramide 10 mg perorally. The maximum neck flexion, extension, and left rotation were noted preoperatively.

In the operating theatre, standard monitors (pulse oximetry, noninvasive blood pressure recording, electrocardiography, and capnography) were connected and all patients received injection midazolam 2 mg I.V. and fentanyl 2 *μ*g/kg I.V. three minutes before induction. Patients were induced with propofol 2 mg/kg I.V. and paralysed with atracurium 0.5 mg/kg I.V. Mask-ventilation was initiated with isoflurane 1.5% and nitrous oxide/oxygen for three minutes, following which a single, experienced anesthesiologist inserted the well-lubricated supraglottic airway device. I-gel was introduced by firmly grasping the device, such that the cuff outlet was facing the chin of the patient and the device was gently guided along the hard palate, until definite resistance was felt, as per the manufacturer's recommendations [8]. Insertion of the PLMA was done as per the manufacturer's recommendations, using the index finger digital method. Anaesthesia was maintained with isoflurane and 50 : 50 nitrous oxide : oxygen with a MAC of 1 to 1.3. Size selection of the I-gel and PLMA was based on patient's weight: size 3 for patients less than 50 kg and size 4 for those between 50 and 90 kg for I-gel and size 3 for patients less than 50 kg, size 4 for those between 50 and 70 kg, and size 5 for those between 70 and 100 kg for PLMA. Cuff pressure was maintained at 60 cm of H_2_O for ProSeal LMA in all positions, using cuff pressure monitoring device (PORTEX, Smiths Medical Inc., UK). Appropriate placement of the I-gel and PLMA was assessed by gently squeezing the reservoir bag and observing the end-tidal carbon dioxide waveform, chest movements, and easy passage of the gastric drain tube [9].

If ventilation was inadequate, the following manipulations were allowed: gentle pushing or pulling of the device, chin lift and jaw thrust. The number of attempts required for insertion was recorded and a “failed attempt” was defined as removal of the device from the mouth before reinsertion. A maximum of three attempts before a failure of insertion were recorded, in which case a tracheal tube was inserted for airway management and the patient was excluded from the study. A well-lubricated gastric tube, size 12 French in ProSeal LMA group and 10 French in I-gel group, was inserted through the drain tube. Correct placement of the gastric tube was assessed by auscultation of injected air by epigastric stethoscopy. The gastric tube was left open throughout the surgery.

After confirming correct placement of the device, the effect of various head and neck positions on the device was evaluated. Neutral position was maintained with the external ear canal level with the top of the shoulder and the ear-eye line (from the external ear canal to the superior orbital margin) vertical and then the patient was repositioned in the following positions: maximal extension, maximal flexion, and maximal rotation to the left as noted preoperatively. Each position change started from the neutral position and the depth of insertion of the supraglottic airway device constantly maintained as in the neutral position. The cuff pressure was also maintained at less than 60 cm H_2_O. In each position, peak inspiratory pressure and leak airway pressure were noted at a set tidal volume of 10 mL/kg. Readings were taken one minute after adjustment of the head and neck position. Leak airway pressure (LAW) (oropharyngeal leak pressure/airway sealing pressure) [10, 11] was determined [12] by placing the anaesthesia circle breathing system in bag or manual mode, with the adjustable pressure limiting (APL) valve closed and a fixed gas flow of 3 L/min (GE S/5 anesthesia delivery system). Airway pressure was allowed to increase (but not permitted to exceed 40 cm H_2_O) until it reached equilibrium, that is, until the leak around the cuff reached 3 L/min. The equilibrating airway pressure was recorded as the airway leak pressure. The leak around cuff was detected by any of the methods (an audible noise by listening over the mouth and/or palpable leak around the cuff/auscultation of noise by using stethoscope placed just lateral to thyroid cartilage). The interobserver reliability and accuracy of this measuring system have already been validated [12]. The differences between the mean leak airway pressure and mean peak airway pressure (LAW-PAW) were also calculated, as described in previous studies [8, 13].

Fibreoptic views were noted by an independent observer who was unaware of the study design. Fibreoptic views were obtained by passing a fibreoptic scope through the airway tube to a position 1 cm proximal to the end of the tube and scored using the Brimacombe score [14] (1: vocal cords not seen, 2: vocal cords plus anterior epiglottis seen, 3: vocal cords plus posterior epiglottis seen, and 4: only vocal cords visible). The ventilation score [15] was scored from 0 to 3 based on three criteria: no leakage with an airway pressure of 15 cm H_2_O, bilateral chest excursions with a peak inspiratory pressure of 20 cm of H_2_O, and a square wave capnogram, with each item scoring 0 or 1 point. Thus, if all three criteria were satisfied, the ventilation score was 3. Any adverse event that occurred with change of position that decreased ventilation was recorded. The device was brought back to position where there was no difficulty ventilating, and if ventilation still did not improve, the device was removed and trachea intubated.

### 2.1. Statistics

A sample size calculation was performed using the OpenEpi Version 2.3.1 software [16], with a confidence interval (2-sided) of 95% and a power of 90%, based upon previous [15, 17] studies. Individual sample sizes for flexion, extension, and lateral rotation between I-gel and ProSeal LMA were calculated. The maximum sample size was 31 in each group for flexion with a difference between means of 4 cm of H_2_O for oropharyngeal leak pressures. We enrolled 62 patients (31 in each group). SPSS Version 20 (IBM Inc., NY, USA) was used for statistical analysis. Continuous measurements were expressed as mean ± standard deviation (SD). Oropharyngeal leak pressures, peak airway pressures, expired tidal volume, and EtCO_2_ were analyzed using the paired *t*-test within the group and unpaired *t*-test between the groups. Brimacombe scores and ventilation scores were compared using the Mann-Whitney test between groups and Wilcoxon test within the groups. *P* < 0.05 was considered significant.

## 3. Results

**Figure 1:**
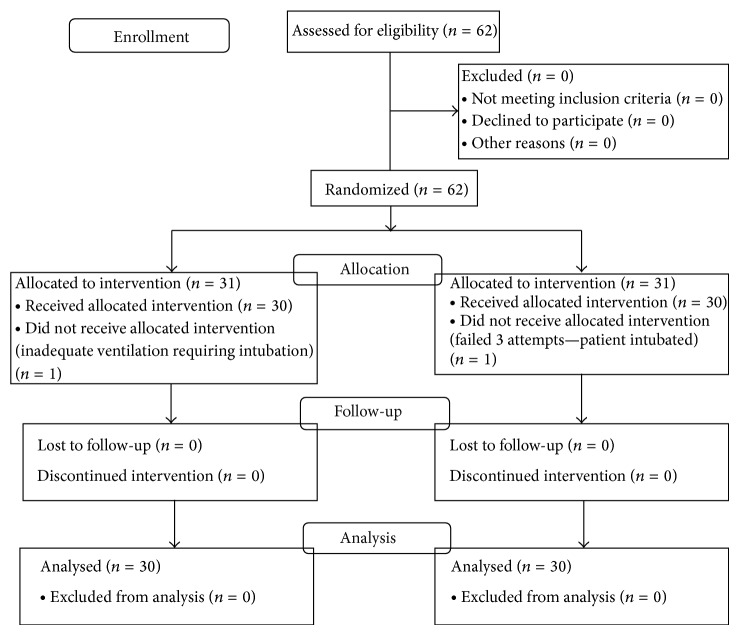
CONSORT figure representing enrolment data.

Figure 1 represents the enrolment data for this study. One patient in each group was excluded from the study because of inadequate ventilation (both patients intubated). CONSORT figure represents enrolment data (Figure 1). Descriptive details of patients are shown in Table 1. Both groups were comparable with regard to demographic characteristics. All devices were inserted in the first attempt.

In the ProSeal LMA group, compared with neutral position, oropharyngeal leak pressures were significantly higher with flexion and lower with extension but similar with rotation of head and neck. Peak airway pressures were significantly higher with flexion and lower with extension but did not change significantly with rotation of head and neck. Tidal volume delivery was comparable in all positions. Sealing pressures decreased significantly with extension (Tables 2 and 6).

In the I-gel group (Tables 4 and 6), compared with neutral position, oropharyngeal leak pressures were significantly higher with flexion and lower with extension but similar with rotation of head and neck. Peak airway pressures were significantly higher with flexion and comparable with extension and did not change significantly with rotation of head and neck. Tidal volume delivery was comparable in all positions. There was a significant reduction in sealing pressures with extension.

## 4. Discussion

The primary objectives of this study were to compare the oropharyngeal leak pressures of I-gel and ProSeal LMA at different head and neck positions, namely, neutral, flexion, extension, and left rotation. We also assessed and compared the fiberoptic view of glottis and ventilator score of both devices in different head and neck positions (secondary objectives). In this study we have demonstrated that the oropharyngeal leak pressure was clinically higher with ProSeal LMA as compared to I-gel in all the positions (Table 6). The peak airway pressure was comparable in both groups in different positions. The difference between leak airway and peak airway (LAW-PAW) pressure was consistently better with ProSeal LMA as compared to I-gel (Table 8) indicating that the former provides wider safety margin for ventilation [8, 13]. To ventilate safely with a laryngeal mask, it is important to use a mask with a high leak pressure and positive pressure ventilation with a lower peak inspiratory pressure [18]. The oropharyngeal leak pressures increased significantly for both ProSeal LMA and I-gel in flexion (Tables 2, 4, 6, and 8). This is also accompanied by a significant increase in airway pressures. However ventilation was maintained as shown by comparable tidal volume exchange and EtCO_2_. The fiberoptic score (as assessed by the view of the glottis) decreased in flexion in both groups but did not affect ventilation. The oropharyngeal leak pressures decrease significantly for ProSeal LMA and I-gel in extension, with a significant decrease in airway pressures in the former (Table 8). However ventilation was maintained in extension position. Lateral rotation did not significantly affect the oropharyngeal leak pressure in both ProSeal and I-gel groups.

The oropharyngeal leak pressures increase significantly for both ProSeal LMA and I-gel in flexion (Tables 2, 4, 6, and 8) (ProSeal LMA: neutral 28 ± 4.19 cm H_2_O and flexion 32 ± 4.11 cm H_2_O and I-gel: neutral 22 ± 3.23 cm H_2_O and flexion 25 ± 3.64 cm H_2_O (Table 6)). This is accompanied by a significant increase in airway pressures, indicating obstruction. However the obstruction did not clinically affect the ventilation, as it did not change the delivered tidal volume or EtCO_2_ significantly. This finding correlates with the results of the study done by Park et al. [17] (neutral 26 ± 6.6 cm H_2_O and flexion 32 ± 5.9 cm H_2_O for ProSeal LMA).

The fiberoptic score frequently decreased in flexion (Tables 3, 5 and 7) but did not affect ventilation as shown by adequate tidal volumes delivered and a comparable end-tidal CO_2_. The finding of epiglottis within the cuff is commonplace [18–20] and does not affect ventilation. The value of fiberoptic position as a means of assessing anatomic position has been questioned [9, 21, 22]. However we assessed the fiberoptic view of the glottis to additionally rule out folding of the epiglottis. Flexion adversely affected ventilation in one case with the ProSeal LMA, with ventilation score of zero and delivered tidal volume of 50 mL. Fiberoptic assessment revealed epiglottis falling backwards and obstructing the airway. The problem was rectified by bringing the head back to neutral position. Nandi et al. [18] have suggested radiological examination (MRI) to identify the exact site of obstruction. Isserles and Rozenberg [6] suggested that neck flexion removes the longitudinal tension in the anterior pharyngeal muscles, allowing them to settle down onto the mask to form a better seal. Neck flexion causes a reduction in the anteroposterior diameter of the pharynx [5].

We also found that the oropharyngeal leak pressures decrease significantly for ProSeal LMA and I-gel in extension, with a significant decrease in airway pressures in the former (Table 8). The tidal volume delivered was comparable and was not compromised. Neck extension increases the anteroposterior diameter by raising the hyoid and the laryngeal inlet. The changes in oropharyngeal leak pressure with flexion and extension are probably unrelated to forces transmitted along the tube.

Flexing the head and neck and avoiding extension may be useful adjuncts to other strategies used to improve seal such as adjusting cuff volume, repositioning the mask, changing size, or applying gentle pressure to the front of the neck.

There was no significant change in the fibreoptic view of the glottis within devices, between positions and between devices, in similar positions. The fibreoptic score frequently decreased in flexion, but the same did not affect ventilation as mentioned earlier.

In this study, lateral rotation did not significantly affect the oropharyngeal leak pressure in both ProSeal and I-gel groups. This is similar to the study done by Sanuki et al. [15], in which I-gel was studied in various head and neck positions (neutral position: 25 ± 5.2 cm H_2_O and rotation: 26 ± 5.1 cm H_2_O), and Park et al. [17] (for ProSeal LMA at neutral position: 26 ± 6.6 cm H_2_O and rotation: 25 ± 5.6 cm H_2_O).

*Limitations of This Study*. (1) The study could not be blinded, as is the case with other similar studies using airway devices as blinding is not possible; however this is unlikely to have skewed the results as the parameters and endpoints were clearly defined. (2) This study was performed in paralysed, anesthetised patients. Therefore, our results may not be applicable to spontaneously breathing patients. (3) Radiological examination (MRI) to identify the exact site of obstruction was not done.

## 5. Conclusion

We conclude that effective ventilation is possible with both ProSeal LMA and I-gel with the head in neutral, flexion, extension, and lateral rotation positions. However, care should be taken with extreme flexion with both ProSeal LMA and I-gel and the airway pressures need to be monitored. ProSeal LMA has a better margin of safety than I-gel due to better airway sealing pressures, except in flexion where the increase in airway pressure is more with the former. The cuff position does not vary with flexion, extension, or lateral rotation of head and neck with both ProSeal LMA and I-gel. During fibreoptic evaluation of glottis, a lower score was obtained with flexion, but the same did not affect ventilation, which was evident from adequate delivered tidal volumes and comparable levels of end-tidal CO_2_ between the neutral and flexion positions.

## Figures and Tables

**Table 1:** Demographics.

Parameter	ProSeal LMA (*n* = 30)	I-gel (*n* = 30)	*P* value
Age (in years)	38 ± 14.3	38 ± 13.1	0.98
Sex (F : M)	16 : 14	18 : 12	0.60
Height (in cm)	158 ± 7.02	159 ± 8.07	0.85
Weight (in kg)	54 ± 11.08	55 ± 11.05	0.93
Mallampati Class (1/2/3/4)	12/13/5/0	13/13/4/0	0.72
ASA physical status (1/2)	14/16	22/8	0.03
Size of device inserted (3/4)	14/16	17/13	0.44

Data presented as mean ± SD or actual numbers. *P* < 0.05 is considered significant.

**Table 2:** Oropharyngeal leak pressures and ventilation with ProSeal LMA

Parameter	Neutral	Flexion	Extension	Lateral rotation
Oropharyngeal leak pressures (cm H_2_O)	28 ± 4.19	32 ± 4.11^*^	24 ± 4.00^#^	28 ± 3.15
Ventilation score (3/2/1/0)	30/0/0/0	29/0/0/1	30/0/0/0	30/0/0/0
Peak airway pressures (cm H_2_O)	16 ± 2.52	19 ± 6.09^©^	15 ± 2.85^®^	16 ± 2.59
Expiratory tidal volume (mL)	461 ± 68.72	452 ± 100.75	457 ± 66	463 ± 66.26
LAW-PAW	11 ± 5.02	12 ± 6.83	9 ± 5.18^¥^	12 ± 4.27
EtCO_2_ (mm Hg)	31 ± 2.33	31 ± 1.98	31 ± 2.24	31 ± 2.06

Data shown are mean ± SD or numbers. *P* value is in comparison with the neutral position.

^*^ *P* < 0.001 between neutral and flexion, ^#^ *P* < 0.001 between neutral and extension.

^©^ *P* = 0.02 between neutral and flexion, ^®^ *P* = 0.04 between neutral and extension.

^¥^ *P* < 0.001 between neutral and extension.

**Table 3:** Fibreoptic view of the glottis with ProSeal LMA.

Brimacombe score	Neutral	Flexion	Extension	Lateral rotation
4	13	11	15	11
3	12	8	11	12
2	3	6	2	5
1	2	5	2	2
*P* Value	N/A	0.058	0.10	0.10

Data in actual numbers; *P* value in comparison with neutral position; *P* < 0.05 is considered significant.

Head and neck position did not significantly alter the fibreoptic view of the glottis through the ProSeal LMA.

**Table 4:** Oropharyngeal leak pressures and ventilation with I-gel.

Parameter	Neutral	Flexion	Extension	Lateral rotation
Oropharyngeal leak pressures (LAW) (cm H_2_O)	22 ± 3.23	25 ± 3.64^*^	19 ± 2.61^#^	22 ± 2.74
Ventilation score (3/2/1/0)	30/0/0/0	30/0/0/0	29/1/0/0	30/0/0/0
Peak airway pressures (cm H_2_O)	15 ± 2.99	17 ± 5.25^©^	15 ± 3.39	16 ± 3.24
Expiratory tidal volume (mL)	481 ± 48.69	481 ± 52.67	477 ± 50.69	478 ± 49.63
LAW-PAW (cm H_2_O)	6 ± 4.86	7 ± 6.23	3 ± 4.46^®^	6 ± 4.77
EtCO_2_ (mm Hg)	31 ± 2.27	31 ± 2.02	31 ± 2.10	31 ± 2.07

Data shown are mean ± SD or numbers. *P* value is in comparison with the neutral position.

^*^ *P* < 0.001 between neutral and flexion, ^#^ *P* < 0.001 between neutral and extension.

^©^ *P* < 0.001 between neutral and flexion, ^®^ *P* < 0.001 between neutral and extension.

**Table 5:** Fibreoptic view of glottis with I-gel.

Brimacombe score	Neutral	Flexion	Extension	Lateral rotation
4	13	8	11	8
3	15	11	13	15
2	1	8	4	5
1	1	3	2	2
*P* value	N/A	0.31	0.08	0.25

Data in actual numbers. *P* value in comparison with neutral position.

*P* < 0.05 is considered significant.

Head and neck position did not significantly alter the fibreoptic view of glottis through I-gel.

**Table 6:** Oropharyngeal leak pressures between devices.

Parameter	Oropharyngeal leak pressure (cm H_2_O)		*P* value
	ProSeal LMA	I-gel	
Neutral	28 ± 4.19	22 ± 3.23	<0.001
Flexion	32 ± 4.11	25 ± 3.64	<0.001
Extension	24 ± 4.00	19 ± 2.61	<0.001
Lateral rotation	28 ± 3.15	22 ± 2.74	<0.001

Data shown are mean ± SD. *P* < 0.05 is considered significant.

The oropharyngeal leak pressures were significantly higher for ProSeal LMA compared with the I-gel in neutral, flexion, extension, and lateral rotation positions.

**Table 7:** Fibreoptic position between the devices.

Parameter	Fibreoptic view of glottis (4/3/2/1)		*P* value
	ProSeal LMA	I-gel	
Neutral	13/12/3/2	13/15/1/1	0.80
Flexion	11/8/6/5	8/11/8/3	0.57
Extension	15/11/2/2	11/13/4/2	0.41
Lateral rotation	11/12/5/2	8/15/5/2	0.95

Data shown in numbers. *P* < 0.05 is considered significant.

Fibreoptic position was similar between the devices and the changes were insignificant in different positions.

**Table 8:** Ventilation parameters, airway parameters, and Brimacombe scores of the devices.

Ventilation parameters								
Parameter	ProSeal LMA				I-gel			
	Neutral	Flexion	Extension	Lateral rotation	Neutral	Flexion	Extension	Lateral rotation
Delivered tidal volumes (mL)	461 ± 68.72	452 ± 100.75	457 ± 66.28	463 ± 66.26	481 ± 48.69	481 ± 52.67	477 ± 50.69	478 ± 49.63
Peak airway pressures (cm H_2_O)	16 ± 2.52	19 ± 6.09	15 ± 2.85	16 ± 2.59	15 ± 2.99	17 ± 5.25	15 ± 3.39	16 ± 3.24
EtCO_2_ (mm Hg)	31 ± 2.33	31 ± 1.98	31 ± 2.24	31 ± 2.06	31 ± 2.27	31 ± 2.02	31 ± 2.10	31 ± 2.07
LAW-PAW (cm H_2_O)	11 ± 5.02	12 ± 6.83	9 ± 5.18	12 ± 4.27	6 ± 4.86^*^	7 ± 6.23^*^	3 ± 4.46^*^	6 ± 4.77^*^
Ventilation score (3/2/1/0)	30/0/0/0	29/0/0/1	30/0/0/0	30/0/0/0	30/0/0/0	29/1/0/0	30/0/0/0	30/0/0/0

Data shown are mean ± SD or numbers. *P* < 0.05 is considered significant. ^*^ *P* value < 0.01.

(LAW-PAW, compared with similar positions between I-gel and ProSeal LMA.) LAW-PAW was consistently better for ProSeal LMA than I-gel in all positions. Other parameters including peak airway pressures, tidal volume delivery, and ventilation scores were comparable between the two groups in all positions.
